# Therapeutic effects of resveratrol and Omega-3 in mice atherosclerosis: focus on histopathological changes

**DOI:** 10.1186/s12906-023-03899-9

**Published:** 2023-03-17

**Authors:** Shamsi Sadat Mosavi, Soghra Rabizadeh, Amirhossein Yadegar, Sara Seifouri, Fatemeh Mohammadi, Reihane Qahremani, Salome Sadat Salehi, Armin Rajab, Alireza Esteghamati, Manouchehr Nakhjavani

**Affiliations:** Endocrinology and Metabolism Research Center (EMRC), Vali-Asr Hospital, Tehran University of Medical Sciences, Imam Khomeini Hospital Complex, Tohid Squre, P.O Box: 13145-784, Tehran, Iran

**Keywords:** Resveratrol, Omega-3, Atherosclerosis, Histopathology

## Abstract

**Background:**

Resveratrol and omega-3 have been shown to prevent atherosclerosis. However, histopathological changes and their comparison have not been studied well. This study investigated the therapeutic effects of resveratrol and omega-3 in experimental atherosclerosis of mice.

**Methods:**

We divided sixty 6-week-old male C57BL/6 mice into six groups and followed for 10 weeks: (1) standard diet, (2) atherogenic diet, (3) atherogenic diet along with resveratrol from the start of the sixth week, (4) atherogenic diet along with omega-3 from the start of the sixth week, (5) standard diet along with resveratrol from the start of the sixth week, (6) standard diet along with omega-3 from the start of the sixth week.

**Results:**

The mice fed on an atherogenic diet had a larger fat area and a thicker aortic wall thickness than mice fed on a standard diet. The use of omega-3 and resveratrol in the mice with an atherogenic diet resulted in a significantly reduced fat area (*p*-value = 0.003), and resveratrol had a significantly higher effect. Omega-3 or resveratrol induced a significant reduction in aortic wall thickness in mice on an atherogenic diet, and there was no significant difference between them. Among the mice with a standard diet, this study did not observe any significant changes in the fat area or the aortic wall thickness with the consumption of omega-3 or resveratrol.

**Conclusions:**

Resveratrol and omega-3 had a regressive and therapeutic role in atherosclerosis, with a more significant effect in favor of resveratrol.

## Introduction

Atherosclerosis is the primary cause of the cardiovascular disease (CVD), including coronary artery disease (CAD), myocardial infarction (MI), stroke, congestive heart failure, and peripheral artery disease, and is a chronic inflammatory condition. Atherosclerosis is mainly located in the intima of medium to large arteries, mainly at the bifurcation site [1]. All the risk factors attributed to atherosclerosis play a part in exacerbating the underlying inflammatory process [2, 3]. One factor shown to be an early event in the atherosclerosis process is oxidation [4]. Radical oxygen species (ROS) are imperative cellular signaling molecules that lead to vascular lesions and endothelial dysfunction [5]. The turning point of vascular endothelial dysfunction is the imbalance between the production of vascular protective substances and vascular relaxants, which precedes many vascular pathological processes and the onset of cardiovascular diseases [6–8].

The formation of atherosclerotic lesions is characterized by excessive accumulation of cholesterol in the arterial intima [9]. Macrophages play an essential role in all stages of atherosclerosis. Disruption of lipid homeostasis in macrophages in atherosclerosis leads to cholesterol accumulation and foam cell formation [10]. Earlier studies have shown that plaque formation is not just an ongoing process associated with aging. It is a dynamic process that can be slowed down, stopped, or reversed [2, 11]. Current treatments of atherosclerotic and cardiovascular diseases mainly focus on statins, which regress plaque by reducing lipid content [12]. Although resveratrol may be effective in atherosclerosis, it may have additional potential benefits as a polyphenol with many pleiotropic actions. Resveratrol has been shown to have various beneficial biological effects such as anti-inflammatory, antioxidant, anti-glycosylation, anti-cancer, anti-aging, and neuroprotective [13].

*Resveratrol* is a polyphenol that was first found in the roots of Veratrum gandiflorum back in 1939 [14]. This polyphenolic phytoalexin is found in grapes, wine, berries, peanuts, and tea. Resveratrol prevents cardiovascular diseases by inhibiting radical oxygen species (ROS), platelet aggregation, and low-density lipoproteins oxidation [15]. Also, resveratrol elicits a regulatory effect on the metabolism of lipids [16]. In a study performed by Cheng et al. on obese mice with a high-fat diet, the anti-inflammatory and antioxidant effects of resveratrol inhibited the accumulation of lipid droplets in hepatocytes, resulting in the protection of the mice from hepatic steatosis [17]. In another study by Huo et al., the anti-inflammatory effects of resveratrol on diabetic mice with coronary diseases led to pancreatic tissue protection and a lower serum sugar level, resulting in cardiovascular protection [18]. Resveratrol is well tolerated in healthy people and by experimental models without significant side effects [13]. Resveratrol has low bioavailability and rapid metabolism, but despite this, it shows a relevant biological effect that may be due to its conversion/interconversion to sulfonate and glucuronide metabolites and/or its binding/dissociation to plasma proteins, the main routes of resveratrol delivery at target organ sites [13].

*Omega-3* is a polyunsaturated fatty acid (PUFA) with its double bond three atoms away from its methyl terminus [19]. It is mainly known for its anti-inflammatory effects partially due to one of its substrates, eicosapentaenoic acid (EPA) [20]. Also, omega-3 has antithrombotic effects and reduces blood pressure, pulse rate, and triglyceride levels [21]. Two randomized clinical trials (JELIS and REDUCE-IT) that were carried out in 2007 and 2020 demonstrated the potential role of EPA in reducing atherosclerosis [22, 23]. Omega-3 fatty acids act as a substrate for forming a group of lipid mediators that relieve inflammation [24].

Pramaningtyas and Faruqy et al. examined the aorta of diabetic rats using Image J software and reported that exercise did not affect aortic wall thickness [25]. Bonanno et al., using ultra-high-resolution 3D imaging, showed significant differences in average vessel wall thickness of the cardiovascular system in atherosclerotic rats on an atherogenic diet [26].

Although resveratrol has been shown to have various beneficial effects, including effects on atherosclerosis, to the best of our knowledge, no studies have been conducted to show the impact of resveratrol on the histopathology of atherosclerosis. Also, the histopathological effects of omega-3 on atherosclerosis and its comparison with resveratrol are not well studied.

This study explored and compared the potential beneficial effects of resveratrol and omega-3 in the prevention and regression of atherosclerosis. Also, this study investigated histopathological factors, including the aortic wall thickness and the area occupied by fat droplets in the aortic wall.

## Materials and methods

Sixty 6-week-old male *C57BL/6* mice were obtained from the Pasteur Institute of Iran (Tehran, Iran). The experiment complied with ARRIVE guidelines. This study followed the National Research Council’s Guide for the Care and Use of Laboratory Animals. This study was approved by the Research Ethics Committee of Tehran University of Medical Sciences (Approval number: 94–03–191-30,088). All mice were housed in plastic cages with a stainless-steel gird lid and wood shaving scattered on the floor. The vivarium was maintained at 23 °C on a 12-h light-dark cycle with lights off at 7 pm. The mice were acclimatized for 2 weeks and fed a regular commercial mouse diet (Behparvar co, Iran. This company produces food for laboratory animals, including mice). At the start of the trial, the mean mouse weight was 24 ± 2.6 g. Throughout the experiment (10 weeks), the mice were given free access to food and water.

To produce the atherogenic diet, 1.25% cholesterol, 0.5% cholic acid, and 15% fat were added to the powdered standard diet pellets. The components were mixed for 45 min, formed into a dough with double-distilled water, rolled into pellets, and then allowed to dry for 1 day in a dehydrator at 29 °C and an additional 2 or 3 days in a 37 °C room [27, 28]. In addition, the pellets were provided to the animals every week, and the mice were fed fresh food from the refrigerator every day. The mice were also randomly divided into six groups. Ten mice were in each group.

Group 1 (control) had a standard diet for 10 weeks. Group 2 had an atherogenic diet for 10 weeks. Group 3, on the other hand, had an atherogenic diet for 10 weeks along with 20 mg/kg/day resveratrol [29] from the start of the sixth week. Group 4 also had an atherogenic diet for 10 weeks, yet along with 600 mg/kg/day omega-3 [30] from the beginning of the sixth week. However, group 5 had a standard diet for 10 weeks and 20 mg/kg/day resveratrol from the start of the sixth week, and group 6 had a standard diet for 10 weeks along with 600 mg/kg/day omega-3 from the beginning of the sixth week. In this study, mice were given the prescribed diet for 5 weeks to reach a balanced state, and at the end of the fifth week, we started the treatment with resveratrol or omega-3 in the target groups.

The resveratrol - a Trunature Company, USA product - was in the form of soft gels containing 250 mg of this substance. The omega-3 from Zahravi Company (Tabriz, Iran) was also in the form of soft gels containing 360 mg of this compound. Resveratrol and omega-3 were added to the drinking water of the animals. Each mouse consumed about 5 mL of water per day [31].

The mice were weighed weekly. At the end of the trial, the mice were fasted between 8 to 12 hours. Then they were killed by carbon dioxide inhalation [32]. Immediately after euthanasia, about 0.5 and 1.0 mL of blood was collected from each mouse using cardiac puncture. The blood was transferred to a test tube, and then its serum was separated by centrifugation at 1500 r/min for 15 minutes. ELISA analyzed the serum to measure the lipid profile, and LDL/HDL cholesterol ratio was calculated.

Furthermore, heart tissue was examined under a loupe to find the aortic arch. The tissues taken from animals were fixed in 10% neutral buffered formalin, processed with the standard histological method, and the sections were stained with Hematoxylin and Eosin (H&E) [33, 34]. The current study used a simple, accessible, yet accurate method to achieve results and evaluate the aortic wall thickness. Besides that, this study investigated the intensity of fat accumulation by using and presenting a method, including measuring the areas occupied by fat. In other words, the current study tried to evaluate this aspect of atherosclerotic lesions using a method that easily yields logical results. Histopathological changes in the aorta of mice were studied by a microscope equipped with a camera (Tucsen, H series). This study measured the thickness of aortic walls (μm) and the areas occupied by fat (fatty streaks) (μm^2^) by Radical IS Capture Pro software.

The Radical IS Capture Pro software (Radical Scientific Equipment PVT. LTD) can measure the length or distance between any two specified points. Therefore, we were able to measure the thickness of the aorta. This study also determined the range of fat droplets using this software. The average area occupied by fat in all slides of each group is a sample of the fat area of that group.

Data analysis was performed using the SPSS software version 22 for windows (SPSS, Inc.). Results were expressed as the mean ± standard deviation. The six groups were compared using one-way ANOVA. Mean levels of serum lipid profile were calculated. We considered a *p-value* lower than 0.05 statistically significant.

## Results

The stages of progression and regression of the fat area and the aortic wall thickness of the mice are given in Table 1. The mice fed on an atherogenic diet had a larger fat area and a thicker aortic wall than those fed on a standard diet. The largest fat area was observed in mice fed on a drug-free atherogenic diet (1880.0 ± 16.6 μm^2^). Compared to the drug-free atherogenic diet group, the use of omega-3 and resveratrol in the mice fed on an atherogenic diet significantly reduced fat area (1481.7 ± 29.3 and 1007.2 ± 13.3 μm^2^) (*p-value* < 0.001). Also, we observed a significantly higher effect of resveratrol than omega-3 in limiting fat area progression in the atherogenic diet mice (*p-value* < 0.001). Similar to the fat area surface, the thickest aortic wall was found in mice who received a drug-free atherogenic diet (115.6 ± 9.1 μm). Omega-3 or resveratrol consumption significantly reduced aortic wall thickness (100.9 ± 16.1 and 89.6 ± 14.9 μm) among mice fed on an atherogenic diet (*p-value* < 0.05). However, there was no significant difference between the effects of omega-3 and resveratrol on aortic wall thickness in mice fed an atherogenic diet (*p-value* = 0.279).

**Table 1:** Stages of progression and regression of fat area surface and thickness of the aortic wall in this study

	Atherogenic diet			Standard diet			*P*-value
	–	Omega-3	Resveratrol	–	Omega-3	Resveratrol	
Fat area surface (μm^2^)	1880.0 ± 16.6 ^*^	1481.7 ± 29.3 ^*^	1007.2 ± 13.3 ^*^	213.2 ± 10.2 ^#^	207.7 ± 10.5 ^#^	202.8 ± 10.9 ^#^	0.003
Thickness of the aortic wall (μm)	115.6 ± 9.1 ^*^	100.9 ± 16.1 ^$^	89.6 ± 14.9 ^¥^	38.3 ± 5.0 ^#^	37.9 ± 7.4 ^#^	37.2 ± 7.2 ^#^	0.011

Data are presented as mean ± SD

*: *p-value* ≤ 0.05, vs. all groups

#: *p-value* ≤ 0.05, vs. atherogenic diet with or without drug

$: *p-value* ≤ 0.05, vs. all groups except atherogenic diet with resveratrol

¥: *p-value* ≤ 0.05, vs. all groups except atherogenic diet with omega-3

**Fig. 1:**
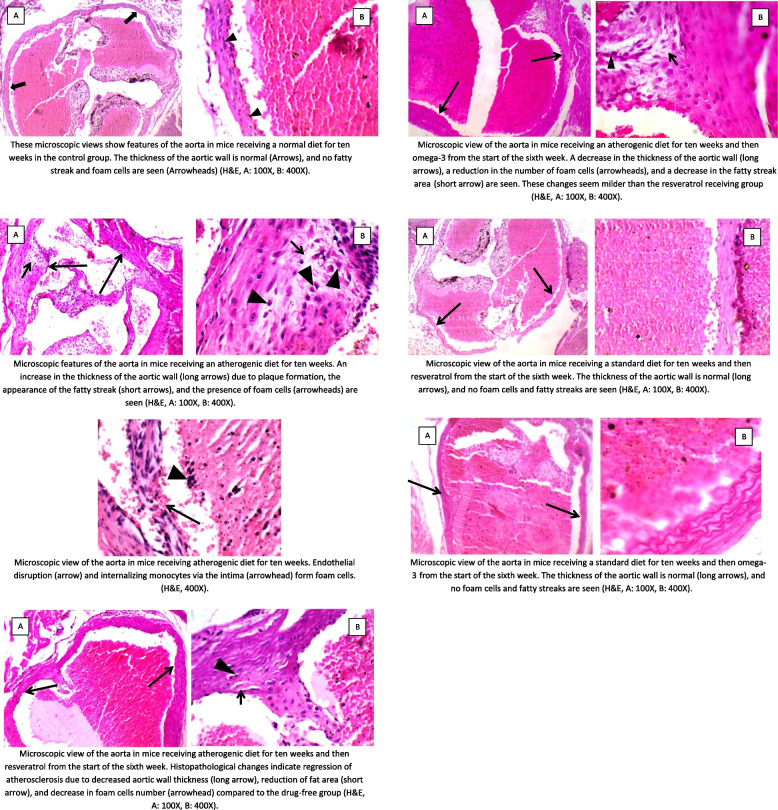
Histopathological changes of the aorta in the studied groups

Among the mice fed on a standard diet, this study did not observe any significant changes in the fat area with the consumption of omega-3 (207.7 ± 10.5 μm^2^) or resveratrol (202.8 ± 10.9 μm^2^) compared to the group with a drug-free diet (213.2 ± 10.2 μm^2^) (all *p-values* > 0.05). Similar results were applied for the thickness of the aortic wall among mice fed on a standard diet (38.3 ± 5.0 μm), standard diet and omega-3 (37.9 ± 7.4 μm), and standard diet and resveratrol (37.2 ± 7.2 μm) (all *p-values* > 0.05). Figure 1 shows the histopathological changes.

**Fig. 2:**
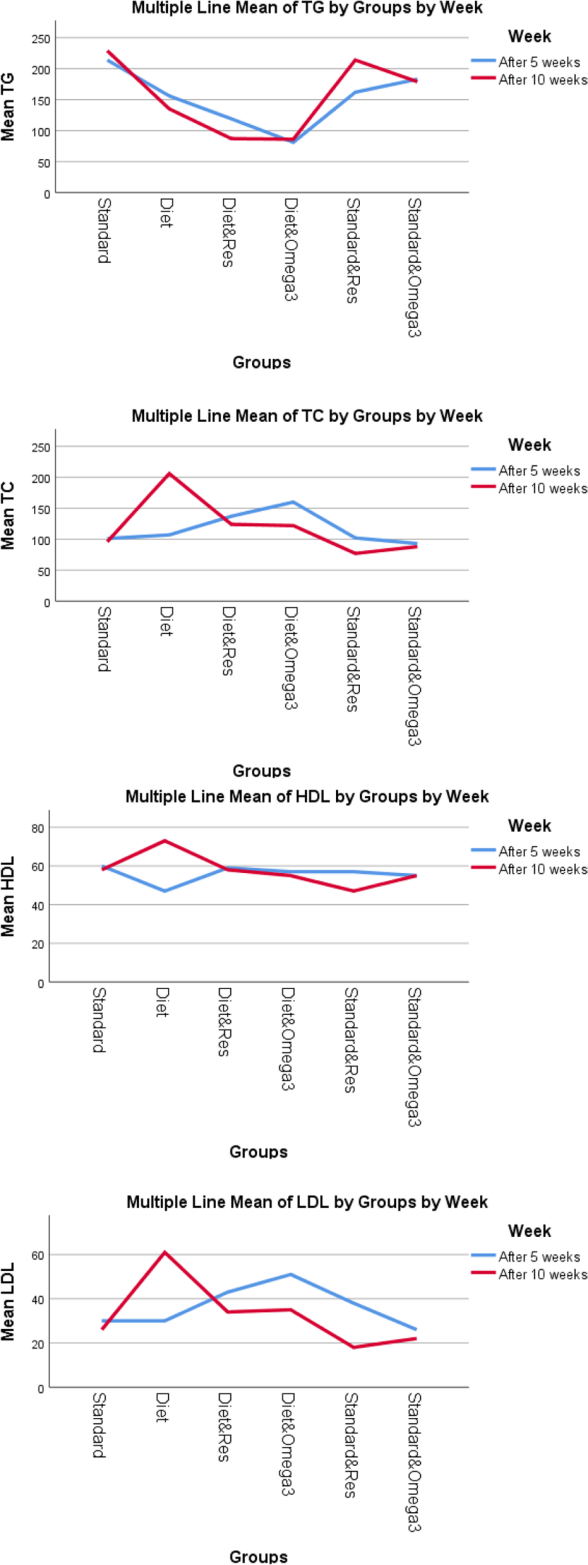
Lipid profile in the studied groups

**Fig. 3:**
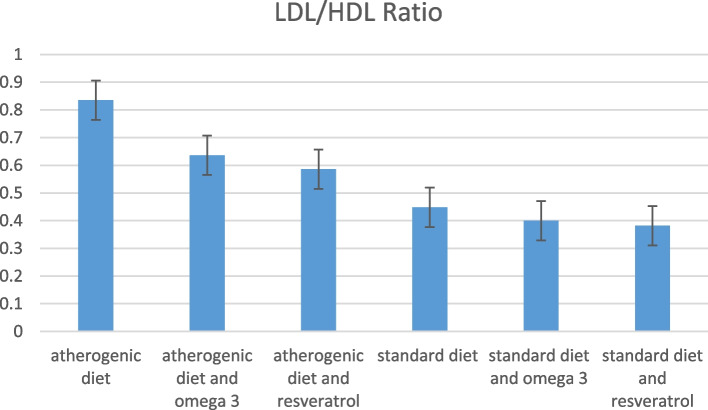
LDL/HDL ratio in the studied groups

Lipid profile components in the studied groups, including TG, TC, LDL-C, and HDL-C levels, are shown in Fig. 2. The mean levels of TC and LDL-C showed a decreasing trend among groups that received treatment (P-trend = 0.532), while mean levels of HDL-C did not change significantly and were relatively stable after 10 weeks (P-trend = 0.315). Furthermore, the LDL/HDL cholesterol ratio was lower in mice receiving treatment, especially resveratrol, either fed on a standard or atherogenic diet (Fig. 3).

## Discussion

This study evaluated the potential effects of resveratrol and omega-3 on atherosclerosis. Features of atherosclerosis, such as incidence of fatty streaks, thickened aortic wall, and increased serum LDL/ HDL ratio, were observed in the mice receiving atherogenic diets in the fifth week but more severe in the tenth week. The area occupied by fat droplets was considered and measured as an indicator of the fatty streak. According to the author’s knowledge, this is the first time that the fat-occupied area in the aortic wall is calculated as a symbol of the fatty streak.

In the current study, resveratrol and omega-3 significantly inhibit the process of atherosclerosis in mice by reducing the area occupied by fat, the aortic wall thickness, and the LDL/HDL ratio. Even in mice receiving standard diets, the addition of treatments reduced fat area, the aortic wall thickness, and the LDL/HDL ratio. Also, resveratrol showed to be more effective than omega-3.

Various strategies have been proposed to prevent and reverse plaque formation, including diet modification, dietary supplements, and medications [20, 24, 35, 36]. Some essential oil compounds, such as eucalyptol, can prevent atherosclerotic lesions in the rat model by decreasing glycation, oxidative stress, and inflammatory mediators [37]. Since 1992, when the cardioprotective effects were attributed to red wine’s moderate and chronic consumption, several studies have been conducted to prove this effect and find its mechanism [38–40]. Resveratrol is a phenolic compound in red wine that has attracted attention for providing an alcohol-free antioxidant [41].

The general mechanisms of the anti-atherosclerotic effect of resveratrol and omega-3 are almost identical. The antioxidant effect mainly exerted by resveratrol is due to increased nitric oxide synthesis [15, 42], while it is exerted by omega-3 further due to reduced production of inflammatory mediators and cytokines [20].

Resveratrol and omega-3 reduce platelet aggregation and inhibit thrombus formation [20, 35, 43]. Reducing the number of macrophages within the plaque [21] and modulating the LDL uptake by them (diminished foam cells) under the influence of omega-3 [44], as well as inhibiting LDL peroxidation and vasorelaxant effect in the influence of resveratrol [15, 16, 43, 45], lead to limited fatty streak and plaque size. Decreased plaque size and the number of foam cells occurred in both treatment groups in this study.

In addition to these similar mechanisms, resveratrol and omega-3 have different and unique mechanisms that may have different effects. Based on the results of this study, resveratrol was able to be more effective than omega-3 in the prevention and the regression of atherosclerosis in mice, which may be due to its phytoestrogenic properties [43] or its ability to increase mitochondrial regeneration and activity [35, 43, 46]. With such a protective effect, resveratrol can be an excellent complementary to statins, which have been mainly used to prevent and treat atherosclerosis [47]. Studies have shown that statins may interfere with mitochondrial activities, and some adverse effects may be caused directly or indirectly by the mitochondrial pathway [48]. In addition, statins stabilize atherosclerotic plaque with thickened fibrous caps and macrocalcifications [12].

Kakoti et al. showed that combination therapy with resveratrol and omega-3 is more valuable, especially in chronic diseases such as Alzheimer’s and atherosclerosis. However, the findings were focused on serum concentrations of inflammatory mediators and not on histopathological parameters. The combined effect was to reduce inflammation by reducing nitric oxide (resveratrol effect) and prostaglandins (omega-3 impact) [35].

In this study, the LDL/HDL ratio (as a marker of atherosclerosis [49]) was significantly increased in mice fed on an atherogenic diet (*p*-value = 0.008). Both resveratrol and omega-3 reduced the LDL/HDL ratio in mice receiving an atherogenic diet. At the same time, neither of these substances significantly affected the LDL/HDL ratio in mice fed on a standard diet.

In the current study, mean levels of TC and LDL-C had a non-significant downward trend among groups receiving treatment. However, after 10 weeks, no significant changes were observed in mean HDL-C levels. The effect of resveratrol or omega-3 on serum lipid profile is highly controversial. Recent in vivo studies have failed to show a significant effect of resveratrol on serum cholesterol levels [43, 50]. However, in some cases, total cholesterol was reduced in resveratrol-treated hypercholesterolemic rats [43, 51]. Penumathsa et al. conducted a study on rats. They showed that the lipid level was decreased in all the treatment groups, more significantly in the statin-treated group when compared to the resveratrol-treated group [42]. The hypocholesterolemic effect of resveratrol may be due to its phenolic hydroxyls, which lead to the oxidation of unsaturated fatty acids and the reduction of circulating cholesterol [52]. Studies reported that omega-3 does not affect serum cholesterol levels [53], while other studies showed that omega-3 could increase HDL [54, 55] or decrease the ratio of total cholesterol to HDL [55, 56]. The increase in HDL-C induced by omega-3 may be explained by increased lipoprotein lipase (LPL) activity [54]. The controversial results may be due to the different conditions of the studies.

There are several limitations in this study:

1. The current study did not investigate the treatment with the combination of resveratrol and omega-3 and its histopathological changes.
2. This study did not look for changes at the cellular scale and did not use monoclonal antibodies and IHC. We only wanted to study histopathological changes and look for an easy way to record changes in aortic wall thickness and fat deposition.
3. Oil Red O staining was not used in this study.

Further studies can investigate the potential effects of combination therapy in atherogenic or standard diet groups. Also, the treatment can be applied for a more extended period. A comparative study between resveratrol and statins may add to the knowledge. Further research is needed, especially clinical trials in humans, to confirm the protective action and demonstrate the clinical aspects of resveratrol.

## Conclusion

In the current study, resveratrol and omega-3 significantly had a regressive and therapeutic role in atherosclerosis, with a more significant effect in favor of resveratrol. More studies are needed to investigate histopathological changes and better understand their mechanism of action.

## Data Availability

The datasets used and/or analyzed during the current study are available from the corresponding author on reasonable request.

## Abbreviations

CAD: Coronary artery disease
CVD: Cardiovascular disease
EPA: Eicosapentaenoic acid
H&E: Hematoxylin and eosin
LPL: Lipoprotein lipase activity
MI: Myocardial infarction
PUFA: Polyunsaturated fatty acid
ROS: Radical oxygen species

## References

[1] Frostegård J (2013). Immunity, atherosclerosis and cardiovascular disease. BMC Med.

[2] Kalanuria AA, Nyquist P, Ling G (2012). The prevention and regression of atherosclerotic plaques: emerging treatments. Vasc Health Risk Manag.

[3] Mallika V, Goswami B, Rajappa M (2007). Atherosclerosis pathophysiology and the role of novel risk factors: a clinicobiochemical perspective. Angiology.

[4] Stocker R, Keaney JF (2004). Role of oxidative modifications in atherosclerosis. Physiol Rev.

[5] Irani K (2000). Oxidant signaling in vascular cell growth, death, and survival: a review of the roles of reactive oxygen species in smooth muscle and endothelial cell mitogenic and apoptotic signaling. Circ Res.

[6] Singh RB (2002). Pathogenesis of atherosclerosis: a multifactorial process. Experiment Clin Cardiol.

[7] Sena C (2018). Vascular oxidative stress: impact and therapeutic approaches. Front Physiol.

[8] Sena CM, Pereira AM, Seiça R (2013). Endothelial dysfunction—a major mediator of diabetic vascular disease. Biochimica et Biophysica Acta (BBA)-Molecular Basis of Disease.

[9] Yu X-H (2019). Cholesterol transport system: an integrated cholesterol transport model involved in atherosclerosis. Prog Lipid Res.

[10] Chistiakov DA, Bobryshev YV, Orekhov AN (2016). Macrophage-mediated cholesterol handling in atherosclerosis. J Cell Mol Med.

[11] Francis AA, Pierce GN (2011). An integrated approach for the mechanisms responsible for atherosclerotic plaque regression. Experiment Clin Cardiol.

[12] Almeida SO, Budoff M (2019). Effect of statins on atherosclerotic plaque. Trends Cardiovasc medicine.

[13] Shaito A (2020). Potential adverse effects of resveratrol: a literature review. Int J Mol Sci.

[14] Springer M, Moco S (2019). Resveratrol and its human metabolites—effects on metabolic health and obesity. Nutrients.

[15] Ramprasath V, Jones P (2010). Anti-atherogenic effects of resveratrol. Eur J Clin Nutr.

[16] Frémont L (2000). Biological effects of resveratrol. Life Sci.

[17] Cheng K (2019). The therapeutic effects of resveratrol on hepatic steatosis in high-fat diet-induced obese mice by improving oxidative stress, inflammation and lipid-related gene transcriptional expression. Med Mole Morphol.

[18] Huo X (2019). Resveratrol effects on a diabetic rat model with coronary heart disease. Med Sci Monitor.

[19] Jenkins DJ (2008). Fish oil and omega-3 fatty acids. Can Med Assoc J.

[20] Simonetto M (2019). A novel anti-inflammatory role of omega-3 PUFAs in prevention and treatment of atherosclerosis and vascular cognitive impairment and dementia. Nutrients.

[21] DiNicolantonio JJ, O’Keefe JH (2020). The benefits of Omega-3 fats for stabilizing and remodeling atherosclerosis. Mo Med.

[22] Yokoyama M (2007). Effects of eicosapentaenoic acid on major coronary events in hypercholesterolaemic patients (JELIS): a randomised open-label, blinded endpoint analysis. Lancet.

[23] Bhatt DL (2020). REDUCE-IT USA: results from the 3146 patients randomized in the United States. Circulation.

[24] Carracedo M (2019). The resolution of inflammation through omega-3 fatty acids in atherosclerosis, intimal hyperplasia, and vascular calcification. Seminars in immunopathology.

[25] Pramaningtyas M, Faruqy M (2021). The aortic wall thickness of diabetes mellitus rat models after routine physical exercise. Atherosclerosis.

[26] Bonanno G (2015). Ultra-high-resolution 3D imaging of atherosclerosis in mice with synchrotron differential phase contrast: a proof of concept study. Sci Rep.

[27] Getz GS, Reardon CA (2006). Diet and murine atherosclerosis. Arterioscler Thromb Vasc Biol.

[28] Vergnes L (2003). Cholesterol and cholate components of an atherogenic diet induce distinct stages of hepatic inflammatory gene expression. J Biol Chem.

[29] Zhang H (2010). Resveratrol improves left ventricular diastolic relaxation in type 2 diabetes by inhibiting oxidative/nitrative stress: in vivo demonstration with magnetic resonance imaging. Am J Phys Heart Circ Phys.

[30] Pisaniello A (2017). Omega-3 fatty acids reduce acute vascular inflammation but do not affect atherosclerotic plaque burden or composition. Heart Lung Circulation.

[31] Bachmanov AA (2002). Food intake, water intake, and drinking spout side preference of 28 mouse strains. Behav Genet.

[32] Underwood W, Anthony R. AVMA guidelines for the euthanasia of animals: 2020 edition. Retrieved on March. 2020;2013(30):2020-1.

[33] Jiang F (2011). Ligustrazine improves atherosclerosis in rat via attenuation of oxidative stress. Pharm Biol.

[34] Bauters D (2018). Functional role of ADAMTS5 in adiposity and metabolic health. PLoS One.

[35] Kakoti BB (2015). Resveratrol and omega-3 fatty acid: its implications in cardiovascular diseases. Front Cardiovasc Med.

[36] Badimon JJ (1989). High density lipoprotein plasma fractions inhibit aortic fatty streaks in cholesterol-fed rabbits. Laboratory Investigation.

[37] Mahdavifard S, Nakhjavani M. Preventive effect of eucalyptol on the formation of aorta lesions in the diabetic‑atherosclerotic rat. Int J Prev Med. 2021;12:45. 10.4103/ijpvm.IJPVM_319_19 PMC8223915 34211676

[38] Lamuela-Raventós RM, Estruch R (2016). Mechanism of the protective effects of wine intake on cardiovascular disease. Wine safety, consumer preference, and human health.

[39] Golan R, Gepner Y, Shai I (2019). Wine and health–new evidence. Eur J Clin Nutr.

[40] Nova E (2019). Wine and beer within a moderate alcohol intake is associated with higher levels of HDL-c and adiponectin. Nutr Res.

[41] Chassot LN (2018). Comparison between red wine and isolated trans-resveratrol on the prevention and regression of atherosclerosis in LDLr (−/−) mice. J Nutr Biochem.

[42] Penumathsa SV (2007). Statin and resveratrol in combination induces cardioprotection against myocardial infarction in hypercholesterolemic rat. J Mol Cell Cardiol.

[43] Baur JA, Sinclair DA (2006). Therapeutic potential of resveratrol: the in vivo evidence. Nat Rev Drug Discov.

[44] Chang CL, Deckelbaum RJ (2013). Omega-3 fatty acids: mechanisms underlying “protective effects” in atherosclerosis. Curr Opin Lipidol.

[45] Leonard SS (2003). Resveratrol scavenges reactive oxygen species and effects radical-induced cellular responses. Biochem Biophys Res Commun.

[46] Galiniak S, Aebisher D, Bartusik-Aebisher D (2019). Health benefits of resveratrol administration. Acta Biochim Pol.

[47] Vaughan CJ, Gotto AM, Basson CT (2000). The evolving role of statins in the management of atherosclerosis. J Am Coll Cardiol.

[48] Mollazadeh H (2021). Effects of statins on mitochondrial pathways. J Cachexia Sarcopenia Muscle.

[49] Enomoto M, et al. LDL-C/HDL-C ratio predicts carotid intima-media thickness progression better than HDL-C or LDL-C alone. J Lipids. 2011;2011:549137. 10.1155/2011/549137 PMC3136137 21773051

[50] Wang Z (2002). Effects of red wine and wine polyphenol resveratrol on platelet aggregation in vivo and in vitro. Int J Mol Med.

[51] Kollar P (2000). Experimental study of resveratrol and flavonoids in red wine with regard to their possible hypolipemic effects. Vnitrni LekarstVi.

[52] Xie H (2014). A study on the effect of resveratrol on lipid metabolism in hyperlipidemic mice. Afr J Traditional Complement Alternative Med.

[53] Goh Y (1997). Effect of w3 fatty acid on plasma lipids, cholesterol and lipoprotein fatty acid content in NIDDM patients. Diabetologia.

[54] Yanai H (2018). An improvement of cardiovascular risk factors by omega-3 polyunsaturated fatty acids. J Clin Med Res.

[55] Zibaeenezhad MJ (2017). Comparison of the effect of omega-3 supplements and fresh fish on lipid profile: a randomized, open-labeled trial. Nutrition Diabetes.

[56] Bradberry JC, Hilleman DE (2013). Overview of omega-3 fatty acid therapies. Pharmacy Therapeutics.

